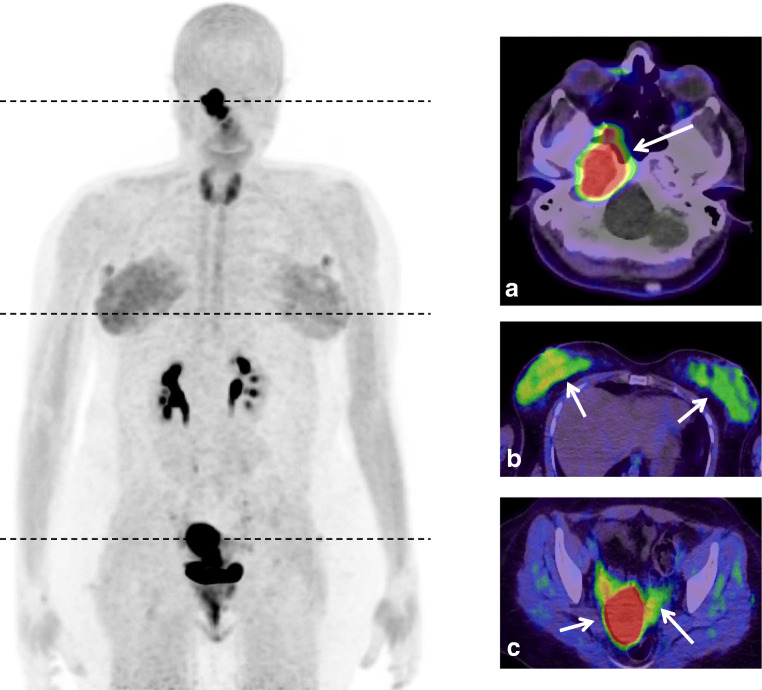# Physiological FAP-activation in a postpartum woman observed in oncological FAPI-PET/CT

**DOI:** 10.1007/s00259-021-05203-8

**Published:** 2021-02-04

**Authors:** Katharina Dendl, Stefan A. Koerber, Sebastian Adeberg, Manuel Röhrich, Clemens Kratochwil, Uwe Haberkorn, Frederik L. Giesel

**Affiliations:** 1 Department of Nuclear Medicine, University Hospital Heidelberg, Heidelberg, Germany; 2 Department of Radiation Oncology, Heidelberg University Hospital, Heidelberg, Germany; 3 Heidelberg Institute of Radiation Oncology (HIRO), Heidelberg, Germany; 4 National Center for Tumor diseases (NCT), Heidelberg, Germany

Fibroblast activation protein (FAP) is a type II transmembrane serine protease overexpressed by cancer-associated fibroblasts [1]. However, high expression can also be observed in non-malignant processes associated with tissue remodeling such as wound healing and diseases leading to fibrosis [2].

Our image presents a 29-year-old female patient with adenoid cystic carcinoma in the right parapharyngeal space with skull infiltration. The tumor was first diagnosed in 12/2018 and subsequently histologically confirmed with an initial classification of cT4 cN0 cM0. Within the scope of planning radiation therapy, a (^68^Ga-FAPI-46) PET/CT was performed presenting a clear finding of the adenoid cystic carcinoma (*a*) with a SUVmax of 17.3, respectively. Surprisingly, the parenchyma in both breasts was well depicted with a SUVmax of 4.1 in the right and 3.5 in the left, respectively (*b*). The endometrium remarkably demonstrated a SUVmax of 25.7 as highlighted in *c*. Uptake in the thyroid (SUV 6.3) was also increased.

We hypothesize that the presented high FAP-uptake in the hormone-sensitive organs such as breast parenchyma and endometrium is a physiological mechanism due to the fact that the presented patient is currently breastfeeding after giving birth 5 months ago. Post-partum thyroiditis is often asymptomatic, thus underdiagnosed and reflects chronic inflammation [3]. Breast parenchyma is characterized by low FAP expression in normal tissue, but high expression in breast cancer [4, 5]. Furthermore, the density of breast and fibro-glandular tissue depends on dynamic physiologic processes based on endogenous and exogenous hormonal fluctuations. During lactation, the cessation of progesterone secretion leads to the release of prolactin, enabling cell vacuolization and secretory changes resulting in the production of milk [6]. Therefore, an increased parenchymal breast density can be seen [7] with an increased FAP expression as a functional correlate.

This case demonstrates that a physiologic increase in FAP expression in hormone-sensitive organs such as the breasts and uterus may occur in the post-pregnancy period and breastfeeding.